# Bruton’s tyrosine kinase inhibition for the prevention of anaphylaxis: an open-label, phase 2 trial

**DOI:** 10.21203/rs.3.rs-2757218/v1

**Published:** 2023-04-05

**Authors:** Ragha V. Suresh, Collin Dunnam, Dhananjay Vaidya, Robert A. Wood, Bruce S. Bochner, Donald W. MacGlashan, Melanie C. Dispenza

**Affiliations:** Johns Hopkins University School of Medicine, Department of Medicine, Division of Allergy and Clinical Immunology, Baltimore, MD; Johns Hopkins University School of Medicine, Department of Medicine, Division of Allergy and Clinical Immunology, Baltimore, MD; Johns Hopkins University School of Medicine, Department of Medicine, Division of General Internal Medicine, Baltimore, MD; Johns Hopkins University School of Medicine, Department of Pediatrics, Division of Allergy, Immunology, and Rheumatology, Baltimore, MD; Northwestern University Feinberg School of Medicine, Department of Medicine, Division of Allergy and Immunology, Chicago, IL; Johns Hopkins University School of Medicine, Department of Medicine, Division of Allergy and Clinical Immunology, Baltimore, MD; Johns Hopkins University School of Medicine, Department of Medicine, Division of Allergy and Clinical Immunology, Baltimore, MD

## Abstract

IgE-mediated anaphylaxis is a potentially fatal systemic allergic reaction for which there are no known preventative therapies. Bruton’s tyrosine kinase (BTK) is an essential enzyme for IgE-mediated signaling pathways, and is an ideal pharmacologic target to prevent allergic reactions. In this open-label trial (NCT05038904), we evaluated the safety and efficacy of acalabrutinib, a BTK inhibitor that is FDA-approved to treat some B cell malignancies, in preventing clinical reactivity to peanut in adults with IgE-mediated peanut allergy. After undergoing a graded oral peanut challenge to establish their baseline level of clinical reactivity, all patients then received four standard doses of 100 mg acalabrutinib twice daily and underwent repeat food challenge. The primary endpoint was the change in patients’ threshold dose of peanut protein to elicit an objective clinical reaction. At baseline, patients tolerated a median of 29 mg of peanut protein before objective clinical reaction. During subsequent food challenge on acalabrutinib, patients’ median tolerated dose significantly increased to 4,044 mg (range, 444 – 4,044 mg). 7 of 10 patients tolerated the maximum protocol amount (4,044 mg) of peanut protein with no objective clinical reaction, and the other 3 patients’ peanut tolerance increased between 32- and 217-fold. Three patients experienced a total of 4 adverse events that were considered by the investigators to be possibly related to acalabrutinib; all events were transient and nonserious. These results demonstrate that acalabrutinib pretreatment can achieve clinically-relevant increases in patients’ tolerance to their food allergen, thereby supporting the need for larger, placebo-controlled trials.

## Introduction

Anaphylaxis is an acute, potentially life-threatening systemic allergic reaction which may be caused by foods, medications, or stinging insect venom in allergic individuals^1^. In IgE-mediated anaphylaxis, allergen cross-linking of specific IgE bound to the surface of mast cells and basophils initiates degranulation and release of mediators that cause urticaria, angioedema, bronchospasm, nausea, vomiting, diarrhea, hypotension, and/or shock^2^. Although there are potential therapies under investigation, there are currently no approved therapies that can reliably prevent anaphylaxis^1^. Intramuscular epinephrine administered after the onset of reaction is the only approved treatment that can potentially mitigate mortality from systemic reactions. Unfortunately, even with prompt and comprehensive medical treatment, anaphylaxis can still be fatal. Standard of care entails allergen avoidance, which is not always feasible for food or stinging insect allergies, where accidental exposures can occur. In addition, patients are often intentionally exposed to known allergens during diagnostic and therapeutic procedures including allergen skin testing, food and environmental allergen immunotherapy, and drug desensitizations, all of which carry the risk of a life-threatening reaction. Therefore, there is an unmet need for therapies that can prevent the occurrence and/or reduce the severity of anaphylaxis^3^.

Acalabrutinib (Calquence^®^, Acerta Pharma and AstraZeneca) is a second-generation oral, covalent inhibitor of Bruton’s tyrosine kinase (BTK), an essential enzyme for high affinity IgE receptor (FcεRI) signaling in human mast cells and basophils^4–6^. Acalabrutinib is currently FDA-approved for some B cell malignancies including chronic lymphocytic leukemia, small lymphocytic lymphoma, and mantle cell lymphoma, and is generally well-tolerated with chronic use^7,8^. In preclinical studies, pretreatment of human skin-derived mast cells and basophils with BTK inhibitors for 15 minutes completely prevented IgE-mediated cell activation, degranulation, and de novo cytokine production^9^. Additionally, premedication with two oral human-equivalent doses of acalabrutinib just hours prior to allergen challenge abrogated moderate-severity anaphylaxis in a humanized mouse model, and reduced mortality from severe anaphylaxis in the same mouse model^9^. Clinically, pretreatment of adults with ibrutinib, a first-in-class BTK inhibitor, reduced or eliminated skin puncture test reactivity to food allergens in food-allergic patients with no observed toxicities^10^. We therefore hypothesized that BTK inhibitors would prevent clinical reactivity to food allergen ingestion in allergic patients. Here, we report the results of a Phase 2 trial investigating the safety and efficacy of a 2-day course of acalabrutinib in preventing clinical reactivity to peanut in patients with peanut allergy.

## Results

### Trial design and patient characteristics:

**Figure 1:**
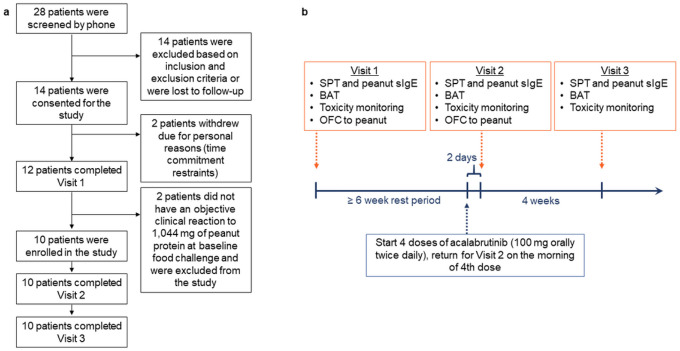
CONSORT flow diagram and study schema. Flow diagram summarizing the (a) enrollment and (b) study visit schedule for the trial. BAT, basophil activation testing; OFC, oral food challenge; sIgE, specific IgE; SPT, skin puncture testing

This prospective, open label trial enrolled adult patients with IgE-mediated peanut allergy (Fig. 1a). Eligible patients underwent baseline placebo-controlled, single-blinded, graded oral food challenge (OFC; **Extended Data Table 1**) to determine their tolerant dose of peanut protein (Fig. 1b). At the first sign of objective clinical reaction (as assessed by using a modified PRACTALL scale^11^; **Extended Data Table 2**), the OFC was stopped and the reaction was treated. After their baseline OFC, patients underwent a rest period of at least 6 weeks prior to starting study drug. All enrolled patients then received 100 mg doses of acalabrutinib by mouth every 12 hours for a total of four doses, returning for repeat OFC on the morning of their last dose. Patients were required to stop all oral antihistamines for at least 7 days prior to study visits, but were permitted to use them and other allergy medications during the remainder of the study. Details of trial design, eligibility criteria, and study procedures are described in the Methods.

A total of 28 patients were screened, of which 14 met eligibility criteria **(Extended Data Table 3**) and were consented (Fig. 1a). Of the 14 patients, 2 withdrew from the study prior to enrollment due to personal reasons. Twelve patients completed a baseline OFC to peanut, afterwards 2 patients were excluded because they failed to meet inclusion criteria. The remaining 10 patients were enrolled and completed the study. Baseline characteristics of the patients are shown in Table 1. Their mean age was 28 years (range, 23 – 36 years); 6 were female sex (60%), 9 were Caucasian (90%), and 3 were Hispanic or Latino (30%).

### Primary Endpoint:

**Figure 2:**
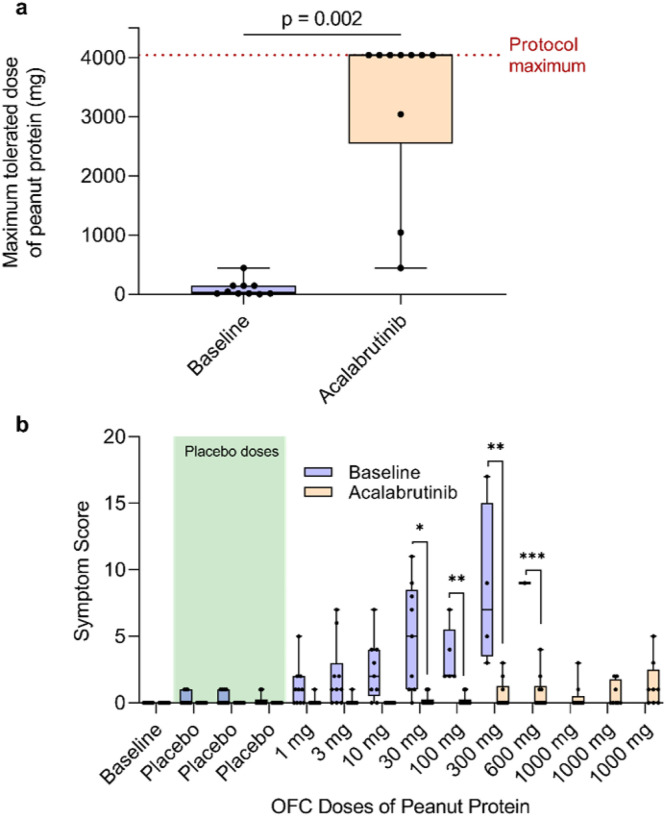
Maximum tolerated peanut dose and symptom scores during OFC. a, The maximum tolerated dose of peanut protein at baseline (dark gray box with blue circles) and during treatment with acalabrutinib (light gray box with orange circles) is shown for all patients (n = 10). The maximum protocol dose was 4,044 mg, thus patients’ tolerated doses of ≥4,044mg on acalabrutinib is plotted at this maximum. Boxplot midline represents median, box depicts 25th and 75th percentiles, and whiskers depict range. Data were tested using a Wilcoxon matched-pairs signed rank test. b, Total symptom scores are shown during each placebo (benign food) and peanut dose during baseline OFC (blue circles and dark gray boxes) and during OFC while on acalabrutinib (orange circles and light gray boxes). The shaded green area represents placebo doses of each OFC. Box plot midline represents the median, box depicts 25th and 75th percentiles, and whiskers depict range. * p < 0.05; ** p < 0.01; *** p < 0.001. OFC, oral food challenge.

The predetermined primary endpoint was the change in patients’ threshold dose of ingested peanut protein to elicit an objective clinical reaction during OFC after acalabrutinib pretreatment compared to patients’ baseline. At baseline, patients tolerated a median of 29 mg of peanut protein (range, 1 – 444) before experiencing an objective clinical reaction during OFC. If needed, all patients were treated with intramuscular epinephrine for their reaction per standard of care in addition to adjunct therapies (e.g. antihistamines or albuterol) at the discretion of the investigator; a summary of rescue medications that were administered to patients during their baseline OFC is listed in **Extended Data Table 4**. During acalabrutinib treatment, patients’ median tolerated dose significantly increased to 4,044 mg (range, 444 – 4,044) of peanut protein (p = 0.002; Fig. 2a). On acalabrutinib, 7 of 10 patients tolerated 4,044 mg, which was the maximum cumulative OFC peanut dose allowed in the study protocol, without having objective clinical reaction or requiring rescue medications (**Extended Data Table 4**). The remaining 3 patients’ tolerance increased from 14 mg of peanut protein at baseline to 444, 1,044, and 3,044 mg with acalabrutinib. These three patients who did not reach the maximum OFC peanut dose while taking acalabrutinib again received rescue medications including intramuscular epinephrine to treat their reactions.

### Secondary Endpoints:

A key secondary endpoint included the change in the severity of clinical reactions during OFC, as assessed by using a modified PRACTALL scale to score symptoms^11^. Symptom scores during OFC were significantly reduced with acalabrutinib therapy at several food doses compared to baseline OFC (Fig 2b). While on acalabrutinib, breakthrough symptoms for the 3 patients that did not reach the maximum amount of peanut included gastrointestinal symptoms (nausea, cramping, and/or diarrhea) in 3 patients, and lower respiratory symptoms (cough, wheezing) in 1 patient. Five of the 7 patients that did tolerate the maximum food challenge dose experienced subjective symptoms during their OFC on acalabrutinib, not considered representative of true positive reactions, which included acid reflux (2 patients), mild nausea(1), mild epigastric pain (1), and scalp pruritus (1).

**Figure 3:**
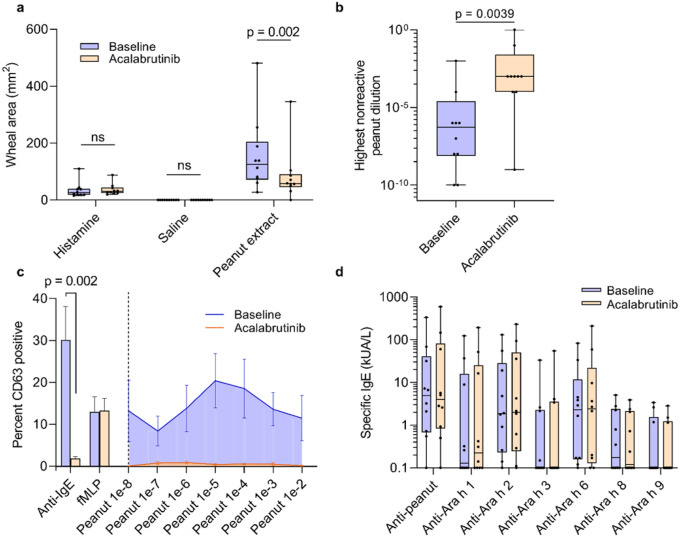
Secondary outcomes a, Skin puncture test wheal area (in mm^2^) to undiluted peanut extract and positive (histamine) and negative (saline) controls at patients’ baseline and during treatment with acalabrutinib are shown for all patients. b, The highest concentration of peanut extract (original units, weight per volume) that produced a negative skin test at baseline and during acalabrutinib treatment is shown for all patients. Boxplot midline represents median, box depicts 25th and 75th percentiles, and whiskers depict range. Data were analyzed using Wilcoxon matched-pairs signed rank tests. c, On the left side of the graph, the percent of basophils activated ex vivo in response to anti-IgE and dilutions are shown for all patients at baseline (blue bars) and after treatment with acalabrutinib (orange bars). On the right, basophil response percentages are displayed for each peanut extract dilution at baseline (blue area under the curve) and after acalabrutinib treatment (orange area under the curve). Data were analyzed using Wilcoxon matched pairs signed rank tests. bars represent standard error. d, Peanut and peanut-component specific IgE levels for all patients at baseline (blue circles) and during acalabrutinib treatment (orange circles) are shown. Box plot midline represents the median, box depicts 25th and 75th percentiles, and whiskers depict range. fMLP, N-formylmethionyl-leucyl-phenylalanine.

Skin puncture testing to peanut extract was included as a secondary endpoint and surrogate marker of mast cell reactivity in vivo. All patients had a positive skin puncture test to undiluted peanut extract at baseline, with a median wheal area of 126 mm^2^ (range, 27.5 – 480). During treatment with acalabrutinib, skin test size to peanut extract was significantly reduced to a median of 57.7 mm^2^ (range, 0 – 345; p = 0.002; Fig. 3a). The trend toward suppression of skin tests was observed at all dilutions of peanut extract (**Extended Data Fig. 1**). When analyzing the highest peanut extract dilution to produce a negative skin test, it was observed that patients’ skin test ‘tolerance’ had increased by a mean of 3.4 ± SEM 0.92 log_10_ units (p = 0.0039; Fig. 3b). Histamine and saline controls were unaffected by acalabrutinib. All skin tests had returned to baseline values size by the third study visit, which was 4 weeks after cessation of acalabrutinib (median 186 mm^2^, range 23.6 – 603; (**Extended Data Fig. 1**).

An additional secondary endpoint was the percentage of basophils activated ex vivo by peanut extract. All patients had a positive basophil activation test (BAT) at baseline to at least one dilution of peanut extract (mean peak response to peanut, 31.7%; range, 3.5 – 70.7; Fig. 3c). While taking acalabrutinib, all patients had completely suppressed basophil activation at all peanut extract dilutions (mean peak response, 1.56%; range, 0 – 3.9; p = 0.002). Anti-IgE responses were also wholly suppressed with acalabrutinib treatment (mean 1.81%; range, 0 – 4.6) compared to baseline (mean, 30.1%; range, 4.3 – 86.7; p = 0.002). All basophil activation responses to anti-IgE antibody and peanut extract returned to baseline values by the third visit (anti-IgE mean, 27.1%, range, 4.5 – 51.1; peanut mean, 28.0%; range, 4.3 – 67.7). Ex vivo basophil activation by N-formylmethionyl-leucyl-phenylalanine (fMLP), a non-IgE-mediated stimulus, was unchanged by acalabrutinib therapy.

### Exploratory Endpoints:

Because BTK plays an important role in B cell receptor signaling and therefore affects plasma B cell survival, exploratory endpoints included markers of humoral immunity function and allergy. All patients had a positive detectable specific IgE to peanut and/or at least one peanut component at baseline, and 9 out of 10 patients had specific IgEs to multiple peanut components (Fig. 3d). No changes in specific IgEs were detected during acalabrutinib therapy (Fig. 3d) or at follow up (**Extended Data Fig. 2**) compared to patients’ baseline. Additionally, levels of quantitative immunoglobulins remained unchanged while on acalabrutinib and at follow up (**Extended Data Table 5**).

### Safety:

Safety endpoints included electrocardiography and laboratory blood testing, including complete blood counts and differentials, serum chemistries, and liver function tests. A total of 15 adverse events occurred in 5 of 10 patients (50%; **Extended Data Table 6**). Four of 15 adverse events were deemed to be at least possibly related to acalabrutinib (summarized in Table 2), which included one grade 2 neutropenia (2.65 to 1.22 K/mm^3^), one grade 1 decrease in hemoglobin (by 0.3 g/dL), and one grade 1 peripheral eosinophilia (from baseline absolute eosinophil count of 250/mm^3^ to 890/mm^3^), which were observed at the second visit and resolved by the third visit. One grade 1 increase in liver function tests was observed at the third visit and was not resolved at study completion because the patient was lost to follow-up. For all adverse events possibly related to acalabrutinib, patients remained otherwise asymptomatic. No patients reported any symptoms while taking acalabrutinib, and none discontinued treatment due to side effects or toxicity. Mean laboratory values across all patients were unchanged with the exception of reduced hemoglobin (mean 13.3 ± 1.60 to 13.2 ± 1.44 g/dL) and increased absolute lymphocyte count (mean 1.99 ± 0.48 to 2.43 ± 0.40 K/mm^3^) while taking acalabrutinib, though both remained within normal laboratory range at all study visits (**Extended Data Table 5**).

Among adverse events not attributed to acalabrutinib, one patient experienced recurrent wheezing after her baseline OFC despite treatment with epinephrine and was sent to the emergency department for further management. Two sports-related concussions occurred during the trial that were considered to be unrelated to study drug or procedures; one occurred prior to receiving acalabrutinib, and one occurred 18 days after the patient’s last dose of acalabrutinib. One of these same patients also experienced a separate mechanical fall due to tripping over an object, which occurred prior to acalabrutinib therapy. No deaths or treatment-related serious adverse events occurred during the trial. No electrocardiographic changes were observed during acalabrutinib treatment.

## Discussion

This trial has demonstrated the first-ever treatment to achieve rapid-onset prevention of IgE-induced anaphylaxis. Results showed that a short course of premedication with standard dosing of the BTK inhibitor acalabrutinib can achieve marked reduction or even complete elimination of clinical reactivity to ingestion of food allergens in allergic patients. Because tolerance to at least 300 mg of peanut protein is considered to be protective against reaction from an accidental exposure^12^, all patients in this trial achieved clinically meaningful increases in their tolerance to peanut. While foods are a very common cause of anaphylaxis^13,14^, regardless of the inducing allergen, all IgE-mediated anaphylaxis in humans is mediated by the FcεRI pathway, of which BTK is an essential kinase. We chose to include peanut-allergic patients in this trial because peanut is one of the most severe food allergies, and there is a strong precedent for utilizing graded OFCs in food allergy trials^13^. Because of shared underlying mechanisms between cases of anaphylaxis, results herein are expected to be applicable for any IgE-mediated systemic allergic reaction.

In parallel with their clinical tolerance, patients’ highest negative skin test dilution of peanut also increased several logs. However, acalabrutinib treatment did not completely suppress skin tests as was observed for basophil activation, which was abolished by acalabrutinib treatment. This is in line with prior studies showing that BTK inhibitor doses that can suppress ex vivo basophil activation responses do not completely inhibit skin tests^10^. The reasons for this disparate activity of BTK inhibitors on identical FcεRI pathways in mast cells versus basophils are as yet unknown. In vitro data suggest that the inhibitory concentrations of BTK inhibitors are similar in human mast cells and basophils^9^. In a previous trial assessing ibrutinib’s effects on skin tests, seven days of ibrutinib did not suppress skin tests further compared to two days^10^. Therefore, one could speculate that longer duration of BTK inhibitor therapy would not offer additional protection against anaphylaxis, and that skin mast cell inhibition in vivo is incomplete at the doses of irreversible BTK inhibitors that are FDA-approved for malignant indications. The dose of acalabrutinib utilized in this trial, which is the FDA-approved dose for treating B cell malignancies, has been shown to result in 100% drug occupancy of BTK in peripheral blood mononuclear cells^15,16^. However, the penetrance of BTK inhibitors into organs such as the skin has not been studied. Full inhibition of tissue-resident mast cells may require different BTK inhibitor dosing than what is used for treating cancers. Interestingly, the 3 patients who did not achieve full protection with acalabrutinib treatment did not demonstrate any symptoms of the skin or mucosa while on acalabrutinib as they had during their baseline OFC.

It is unknown why some patients did not achieve the same magnitude of clinical protection from food-induced anaphylaxis with acalabrutinib treatment as others. Interestingly, these results illustrate the role of mast cells in anaphylaxis, given that some patients still reacted to peanut ingestion despite complete inhibition of their basophils by acalabrutinib. However, no correlation was observed between patients’ clinical response and their baseline skin test size or percent reduction, baseline basophil activation, specific IgE to peanut or components, total IgE or specific-to-total IgE ratios, baseline tryptase level (data not shown), body weight, or body mass index, though our study was not powered to detect such correlations. Further trials are necessary to determine the minimum dose of BTK inhibitors required to attain reliable protection against systemic allergic reactions for all patients.

Given their remarkably rapid onset of action (within days), BTK inhibitors may be a superior choice as adjunct prophylactic therapy for procedures such as high-risk immunotherapy or desensitizations compared with the anti-IgE monoclonal antibody omalizumab, which requires at least 4–8 weeks to achieve an effect and which does not reliably increase tolerance in every patient^17^. Based on in vitro data and animal models, it may be possible to utilize inhibitors of essential kinases such as BTK or spleen tyrosine kinase (SYK) to prevent IgE-mediated reactivity during these procedures without interfering with the allergen desensitization process in mast cells and basophils^18,19^. For example, BTK inhibitors could be administered for brief courses at the onset of food oral immunotherapy to allow patients to reach their maintenance dose without adverse reactions, and discontinued once allergen desensitization (hyporesponsiveness) is achieved. Additionally, though this trial did not assess the recovery time of skin tests and basophil activation after cessation of acalabrutinib, prior human studies have shown that these parameters return to baseline within a week of cessation of covalent BTK inhibitor therapy^10^, suggesting that these medications have a short duration of action, which may be advantageous in some clinical contexts.

Our results, in addition to prior trials utilizing the first-in-class BTK inhibitor ibrutinib to suppress allergen skin tests^10,20^, demonstrate that brief treatment with BTK inhibitors is well-tolerated in healthy patients. Laboratory changes in our study were mild to moderate in severity, largely reversible, and did not cause symptoms or illness in patients. To date, there are no data on the safety of chronic use (months to years) of acalabrutinib in healthy patients without cancers. The most common side-effects of chronic acalabrutinib therapy in patients with cancer include gastrointestinal upset, headache, and infection, along with more rare but serious side-effects including cytopenias, bleeding, arrhythmias, and hypertension^21^. Interestingly, many of these side-effects are thought to be due to off-target effects, and could theoretically be avoided by the use of compounds with higher specificity for BTK. Encouragingly, next-generation BTK inhibitors that are currently in development for chronic urticaria and autoimmune diseases are more selective for BTK with fewer off-target effects, and therefore show more favorable side-effect profiles^22–25^. For example, 12 weeks of remibrutinib (manufacturer: Novartis) was well-tolerated in a phase 2 trial for chronic urticaria with no observed bleeding, arrhythmia, or hypertension events in the treatment arms^22^, as well as during the open-label extension of this trial for up to 52 weeks. Further studies are needed to delineate the safety and utility of prolonged administration of BTK inhibitors before these drugs could be used chronically in healthy patients for allergy indications; for example, to prevent reactivity from an accidental food exposure.

Limitations of our study include a small patient population, the lack of fully blinded OFCs, and the lack of placebo treatment arm. We attempted to mitigate any potential placebo effects or bias by using a modified PRACTALL scale^11^ (**Extended Data Table 4**) to maintain objectivity when assessing symptoms and continuing each OFC until an objective clinical reaction, or the final peanut dose, was achieved. Additionally, this trial did not investigate alternative durations or dosages of acalabrutinib; therefore, the minimum effective duration and dose are as yet undetermined.

In conclusion, we have shown that pretreatment with the oral BTK inhibitor acalabrutinib for just 2 days significantly increases peanut-allergic patients’ tolerance to peanut during oral exposure. These results support the need for larger, placebo-controlled trials to further evaluate the safety and efficacy of these drugs in the context of both short-term and chronic administration to prevent anaphylaxis.

## Online Methods

### Study Design and Oversight:

This trial was conducted under the approval of a United States Food and Drug Administration (FDA) Investigational New Drug application (IND 142734) and the Johns Hopkins University Institutional Review Board (IRB; IRB00223615). The full trial protocol is available in Supplementary information. All study procedures were conducted at a single site (Johns Hopkins University School of Medicine) in accordance with international ethics guidelines and local ethical and legal requirements, including the Declaration of Helsinki. Study visits were completed in the Clinical Research Unit at the Johns Hopkins University Bayview Campus in Baltimore, Maryland after written informed consent was obtained. All study visits occurred between December 2021 and October 2022. Patients stopped all antihistamines and medications with antihistamine properties at least 1 week prior to study visits in preparation for skin puncture testing and oral food challenge (OFC). At Visit 1, medical history, vital signs, height, and weight were collected, and an electrocardiogram was performed (Fig. 1). Patients underwent pregnancy testing (if applicable), skin testing, and basophil activation testing prior to food challenge. They then completed an OFC to peanut to confirm clinical reactivity at baseline. Patients with asthma underwent in-office spirometry prior to beginning OFC to confirm adequate asthma control, which was defined as having a forced expiratory volume in 1 second ≥ 80% of predicted for the patient. Visit 1 was followed by a rest period of at least 6 weeks. At the end of this rest period (and 2 days before Visit 2) patients began treatment with 4 standard oral doses of acalabrutinib (100 mg) every 12 hours. Patients received their fourth and final dose of acalabrutinib on the morning of Visit 2. At Visit 2, patients underwent the same procedures as Visit 1. Four weeks following Visit 2, all patients returned for a follow-up visit (Visit 3) for repeat skin testing, basophil activation testing, and laboratory testing.

### Patient recruitment, screening, and eligibility:

Eligible patients were 18 years of age or older at screening with a history of an IgE-mediated allergy to peanut. Patients were required to have a positive skin puncture test to peanut extract and an objective clinical reaction to cumulative dose of 1,044 mg of peanut protein or less during baseline OFC. Key exclusion criteria included: cardiovascular disease or prior cerebrovascular accident; active infection; history of bleeding disorder or receiving anticoagulants; any immunomodulatory therapies or oral corticosteroids within 1 month prior to study participation; active infection or latent hepatitis; use of strong CYP3A4 inducers or inhibitors; and pregnancy or nursing. Patients were also excluded if they had ever received peanut oral immunotherapy or omalizumab. Complete eligibility criteria are listed in **Extended Data Table 1**.

Patients were recruited from the Johns Hopkins University Allergy and Clinical Immunology outpatient clinic and through IRB-approved advertising on social media. Patients who responded to advertisements were initially screened by telephone to determine eligibility. Patients who were taking proton pump inhibitors were instructed to stop these medications 7 days prior to enrollment. If determined eligible, patients were remote consented prior to Visit 1 by teleconference in compliance with FDA 21 CFR Part11.

### Endpoints:

The predetermined primary endpoint was the change in patients’ threshold dose of ingested peanut protein to elicit an objective clinical reaction during OFC after acalabrutinib pretreatment compared to patients’ baseline. A key secondary endpoint included the change in the severity of clinical reactions during OFC, as assessed by using a modified PRACTALL scale to score symptoms^11^. Other secondary endpoints included size of the skin test wheal to peanut extract and the percent of basophils activated ex vivo by peanut extract while receiving acalabrutinib compared to baseline. Safety endpoints included electrocardiography and laboratory blood testing, including complete blood counts and differentials, serum chemistries, and liver function tests. Exploratory endpoints included changes in circulating quantitative immunoglobulins and serum specific IgE to peanut and peanut components.

### Skin puncture testing:

End-point titration skin puncture testing was performed using whole peanut extract (Greer), undiluted and in 9 serial 1:10 dilutions (original units given by manufacturer, weight/volume). Histamine (1 mg/mL; ALK) and saline (Greer) were used as positive and negative controls, respectively. Lincoln Diagnostics Multi-test devices and testing trays were used for skin testing application for all extract dilutions and controls. Skin tests were read 15 minutes after application. The wheal and flare were each circled with a ballpoint pen and transferred to a clear adhesive sheet, from which the largest diameter and its shortest perpendicular diameter for each wheal was measured in millimeters. Skin test wheal area was calculated as π*(average radius)^2^.

### Oral food challenge to peanut:

All patients underwent a patient-blinded, placebo-controlled, graded OFC to peanut at Visit 1 to establish their baseline level of clinical reactivity to peanut. The food challenge protocol was designed to detect the No Observed Adverse Effect Level, the highest dose observed not to produce any adverse effect, for each patient^26^. While not ideal, it was essential that both placebo and peanut challenges be completed on the same day due to regulatory constraints on the duration of acalabrutinib dosing. To accomplish this, subjects were given 3 varying doses of placebo followed by graded doses of peanut (**Extended Data Table 1**). The amounts of placebo (oat flour; Bob’s Red Mill) or peanut (organic defatted light roast peanut flour; Anthony’s) needed for each dose were calculated based on the target protein amount and the protein content per weight listed on food packaging. Doses were prepared by mixing dry flour with chocolate pudding (Snack Pack) as a vehicle. Visit 1 began with placebo challenge and then immediately continued with peanut food challenge in increasing doses from 1 mg to 1000 mg peanut protein, for a total cumulative goal amount of 4,044 mg of peanut protein (**Extended Data Table 1**). Patients were blinded to peanut or oat doses by using nose clips during dose consumption and coating their mouth in a flavored beverage such as juice or coffee immediately after each dose. Vital signs and physical exam were repeated roughly every 15 minutes throughout the OFC. Symptoms were assessed during OFC by a board-certified allergist/immunologist using a predefined symptom scale (**Extended Data Table 2**; further details can be found below under Symptom Score Assessment). Food doses were given every 15 minutes until a patient had an objective clinical reaction as determined by the symptom scoring scale, at which point the food challenge was stopped, and the reaction was treated using intramuscular epinephrine, plus additional adjunct therapies at the discretion of the investigator (**Extended Data Table 4**). In the event of moderate to severe subjective symptoms alone, the time between food challenge doses was extended, or the food challenge was stopped per the scoring system. Patients continued in the trial if they had an objective clinical reaction by a total cumulative dose of 1,044 mg of peanut protein or less during the Visit 1 OFC. All patients underwent identical repeat OFC at Visit 2 to establish their level of clinical reactivity while taking acalabrutinib.

### Symptom score assessment:

Adapted from the PRACTALL scale^11^, a predefined scoring system was utilized to assess symptoms during OFC and determine clinical reactivity (**Extended Data Table 2**). Symptoms were color-coded to indicate the level of severity and likelihood of their representing true clinical reactivity rather than an anxiety reaction. In brief, green scores included symptoms that did not likely represent a true reaction and were not an indication to stop or delay peanut dosing. Orange scores were judged to be representative of a true reaction if 2 or more orange symptoms recurred for 3 consecutive peanut doses, in which case the food challenge was considered positive, and dosing was stopped. In the case of single, isolated orange symptoms, dosing was continued and/or delayed based on the clinical judgement of the principal investigator. Red scores represented objective symptoms that were highly likely to represent a true clinical reaction; therefore, any red symptom was an indication to stop dosing. Individual symptoms were recorded at each food dose, and combined symptom scores for each dose were calculated.

### Basophil activation testing:

Whole blood samples drawn into 4 mL lithium heparin phlebotomy tubes (BD Biosciences) prior to food challenge at each visit were utilized for basophil activation testing. Whole blood was incubated with mouse IgM anti-human-IgE monoclonal antibody (clone 6061P, Hybridoma Labs), the indicated dilutions of peanut extract (Greer), 1 μM N-formylmethionyl-leucyl-phenylalanine (Sigma), or vehicle (Greer incipient control solution) in PAGCM buffer (piperazine-N,N′-bis[2-ethanesulfonic acid] + bovine serum albumin [MP Biomedicals] + glucose [Sigma-Aldrich] + 1.7 mM calcium + 1.7 mM magnesium) for 30 minutes at 37 °C. Cells were then fixed using Phosflow Fix Buffer (BD Biosciences), centrifuged at 400 × g for 5 minutes, and resuspended in Pipes buffer with 1 mM EDTA and 0.25% bovine serum albumin. Cells were blocked with 1 mg/mL nonspecific human IgG (MB Biological) and then incubated with the fluorescently-conjugated monoclonal antibodies anti-CD63 (1/1000, BD-Pharmingen) and anti-FcεRIα (clone CRA-1, 1/250, Life Technologies) for 25 minutes at room temperature, then with secondary antibodies anti-CD123-PE (1/100, BD Biosciences), anti-mouse IgG2b-AlexaFluor488 (1/1000, Life Technologies), and anti-mouse IgG1-AlexaFluor647 (1/1000, Life Technologies) for 25 minutes at room temperature before analysis on a BD Accuri C6 flow cytometer. The percentage of CD63 positive cells was recorded for each sample. All conditions were normalized by subtracting the percent activation in vehicle-treated samples; in the event of a negative value from this normalization, the result was recorded as zero. The highest percent of basophil activation obtained from three anti-IgE concentrations (0.1, 1, or 10 μg/mL) was reported as the maximum IgE-mediated stimulation for that sample.

### Laboratory testing and toxicity monitoring:

All patients underwent medical interview, physical exam, and laboratory testing at all visits to monitor for safety. Laboratory testing for toxicity monitoring (complete blood counts, serum chemistries, and quantitative immunoglobulins) was performed at each visit prior to OFC by the Johns Hopkins Core Pathology Laboratory. Adverse event determinations were made using the FDA Guidance for Industry: Toxicity Grading Scale for Healthy Adult and Adolescent Volunteers Enrolled in Preventive Vaccine Clinical Trials. Hepatitis serologies were also performed at Visit 1. Prior to the start of the food challenges, laboratory quantification of total IgE and specific IgE to peanut and peanut components were performed using the Phadia ImmunoCAP^™^ platform by the Johns Hopkins Dermatology, Allergy, and Clinical Immunology Reference Laboratory (DACI). The lower limit of detection for the Phadia system is 0.1 KUA/L for all specific IgEs.

### Statistics:

All null hypothesis significance tests were two-tailed. Normality testing was performed on all data using the Shapiro-Wilk test (alpha = 0.05). In the event that data did not demonstrate normal Gaussian distribution, non-parametric tests were utilized as described. For tests using multiple comparisons, corrections for multiplicity were employed as described, and only the adjusted p values are presented in the manuscript. In analyses where there was a significant dose by treatment interaction at the p = 0.05 level, post-hoc differences were presented separately for each dose using the Wilcoxon matched pairs signed rank test. All data were analyzed using Graphpad Prism software, version 9.2.0.

All patients who received acalabrutinib (n = 10) were included in the data analyses. One blood sample was misplaced by the core pathology laboratory, resulting in missing data for Patient 006’s complete blood counts at Visit 2; this was considered to have been a random occurrence, and sampling could not be repeated based on the timing of the sample draw. Basophil activation data were lost for Patient 008’s Visit 3 due to cytometer malfunction; this was also considered to have been a random occurrence. Due to the randomness of these 2 individual events, no statistical adjustments were made.

Sample size was determined pre-trial based on the primary outcome, the change in patients’ threshold dose of peanut to induce clinical reactivity while taking acalabrutinib as compared to baseline. We estimated that 10 subjects allowed for 80% power to detect a 3-fold increase (1.1 natural log units; i.e. 1 food dose escalation) in the threshold food dose using a paired *t* test with p<0.05. For this sample size determination, the primary endpoint was assumed to be normally distributed with a standard deviation of1.1 natural log units. Because most patients tolerated the highest cumulative amount of peanut during food challenge after acalabrutinib treatment, the primary outcome was analyzed as a censored variable post-trial using a Wilcoxon matched-pairs signed rank test with p < 0.05.

For symptom scores, two-way RM ANOVA with Geisser-Greenhouse correction was used to determine interaction between treatment (baseline versus acalabrutinib), food challenge dose, and patient on total symptom scores at each food challenge dose and an interaction effect of treatment by challenge dose. Once a patient displayed an objective clinical reaction, symptom scoring was ceased. For statistical analysis only, the final symptom score value was duplicated for the remainder of (unconsumed) food doses in order to perform ANOVA. This adjustment is not reflected in the graph in Figure 1, where symptom scores are not displayed after the final tolerated food dose. For multiple comparisons between baseline and acalabrutinib at each food challenge dose, Šídák’s multiple comparisons correction was used, with individual variances computed for each comparison, and an alpha threshold of 0.05.

Due to non-normal distribution of skin puncture testing data, the significant interaction effect between treatment and extract dilution, and that many skin tests showed no wheal response at lower concentrations of peanut extract, skin test size to undiluted peanut extract was analyzed separately using a Wilcoxon matched-pairs signed rank test. The highest non-reactive skin test was also analyzed using a Wilcoxon matched-pairs signed rank test (p < 0.05) due to its non-normal distribution.

Because the interaction effect between treatment and extract dilution was significant, and at lower concentrations of extract, many basophil activation responses were 0, peanut extract dilution responses were analyzed using the area under the curve. The mean response for each patient across all peanut extract dilutions was calculated and analyzed using Wilcoxon matched-pairs signed rank test to compare baseline to acalabrutinib treatment. This mean height was not multiplied by width given that the peanut extract has only relative units (w/v). Peanut response means were graphed as area under the curve. This test was also used to compare responses to anti-IgE and N-formylmethionyl-leucyl-phenylalanine.

To analyze peanut- and peanut component-specific IgE values, a two-way RM ANOVA with Geisser-Greenhouse correction was used to determine interaction between treatment (baseline versus acalabrutinib), peanut (or component), and patient on the level of specific IgE. Multiplicity-adjusted p values were calculated using a family-wise alpha threshold of 0.05.

Other laboratory values obtained for safety analysis were analyzed with one-way ANOVA with the Geisser-Greenhouse correction when applicable (for serum chemistries and quantitative immunoglobulins), and otherwise were analyzed with a mixed-effects analysis (for complete blood counts).

### Research reporting:

Further information on study design is available in the Nature Portfolio Reporting Summary linked to this article.

## Figures and Tables

**Table 1:** Baseline characteristics of patients

Characteristic	Total (N = 10)
Age – Median (range)	28 (23–36)
Female sex* – no. (%)	6 (60%)
Race – no. (%)	
White	9 (90%)
African American	1 (10%)
Ethnicity – no. (%)	
Hispanic or Latino	3 (30%)
Not Hispanic or Latino	7 (70%)
Atopic comorbidities – no. (%)	
Allergy to other foods	7 (70%)
Tree nuts	6 (60%)
Soy	2 (20%)
Other legumes	1 (10%)
Asthma	7 (70%)
Rhinitis	9 (90%)
Atopic dermatitis	2 (20%)
Approximate time since last accidental exposure to peanut in months – Median (range)	32.5 (9–72)
History of epinephrine autoinjector use for peanut exposure – no. (%)	
Yes	6 (60%)
No	2 (20%)
Unsure	2 (20%)
History of emergency room visit for peanut exposure – no. (%)	8 (80%)
History of hospitalization for peanut exposure – no. (%)	2 (20%)
Baseline maximum tolerated peanut dose during oral food challenge in mg – Median (range)	29 (1–444)
Peanut extract skin puncture test wheal area in mm^2^ – Median (range)	125.7 (27.5–480.7)
Serum specific IgEs in kUA/L – Median (range)**	
Peanut	4.9 (0–335.0)
Ara h 1	0.13 (0–123.0)
Ara h 2	1.9 (0–131.0)
Ara h 3	<0.1 (0–33.2)
Ara h 6	2.3 (0–82.7)
Ara h 8	0.18 (0–5.1)
Ara h 9	<0.1 (0–3.4)
Total IgE in kU/L – Median (range)	117.0 (18.6–2939)

* One patient is transgender (assigned female sex at birth, currently identifying as male)

** Limit of detection was 0.1 kUA/L

**Table 2:** Summary of adverse events

Category	Total
All adverse events – no.	15
Treatment-related adverse events* – no.	4
Neutropenia	1
Decreased hemoglobin	1
Elevated liver function tests	1
Peripheral eosinophilia	1
Patients with ≥1 treatment-related adverse event – no. (%)	1 (10%)
Grade 4 adverse events – no.	4
Sports-related concussion	2
Sports-related fall and injury	1
Emergency room visit after baseline food challenge	1
Treatment-related serious adverse events – no.	0
Patients who discontinued treatment or study because of an adverse event – no. (%)	0 (0%)

* Adverse events that were assessed as being “possibly” or “probably” related to treatment by the study investigator were recorded as being related to the study drug.

## Data Availability

The data that support the findings of this study are available from the corresponding author upon reasonable request. All requests must be clearly described in writing. All shared data will be de-identified according to applicable regulations.
